# Bioelectrical Impedance in Monitoring Hyperhydration and Muscle Wasting in Critically Ill Corona Virus Disease (COVID-19) Patients: The Feasibility of Predicting Outcome

**DOI:** 10.33549/physiolres.935748

**Published:** 2025-12-01

**Authors:** Marcela KÁŇOVÁ, Karin PETŘEKOVÁ, Nadezhda BORZENKO, Klára RUSKOVÁ, Ivana NYTRA, Pavla DZURŇÁKOVÁ, Michal BURDA, Jan KONVIČKA

**Affiliations:** 1 Institute of Physiology and Pathophysiology, Faculty of Medicine, University of Ostrava, Czech Republic; 2 Department of Anesthesiology and Intensive Care Medicine, University Hospital Ostrava, Czech Republic; 3 Department of Anesthesiology and Intensive Care Medicine, Faculty of Medicine, University of Ostrava, Czech Republic; 4 Institute for Research and Applications of Fuzzy Modeling, University of Ostrava, CE IT4Innovations, Czech Republic

**Keywords:** Bioelectrical impedance, Hyperhydration, Muscle mass, Critically ill patients, Mortality

## Abstract

Critically ill patients often experience hyperhydration and muscle wasting, which can worsen outcomes. This study evaluated the feasibility of using bioelectrical impedance vector analysis (BIVA) to monitor hydration and muscle mass and predict outcomes in COVID-19 patients with acute respiratory distress syndrome (ARDS), including those with extracorporeal membrane oxygenation (ECMO). The study compare fluid parameters derived from BIVA with cumulative fluid balance (CFB) and assess the prognostic value of the phase angle (PA) of BIVA against established markers such an APACHE II and serum presepsin. In this prospective, blinded observational study, 61 COVID-19 patients on invasive mechanical ventilation (IMV) were included. BIVA measurements were taken within 48 h of admission, then after 7 and 14 days. Data on demographics, fluid balance, and laboratory markers were collected. BIVA was shown to be feasible in critically ill patients, with a significant correlation between hyperhydration, defined by an elevated extracellular water to total body water ratio (ECW/TBW 0.56) and overhydration (OHY 6.9 l). Decreased PA (median 3.3°) was associated with increased mortality in non-ECMO patients. Unlike CFB, which lacked statistical significance, BIVA provided a more accurate assessment of hyperhydration (p=0.0050 for ECW/TBW and p=0.0402 for OHY). In conclusion, BIVA is a practical tool for monitoring hydration, but not muscle mass, in critically ill patients. Elevated hydration status and low PA measured by BIVA are effective predictors of mortality, although ECMO use can affect accuracy. ClinicalTrials.gov ID NCT04758676 (www.clinicaltrials.gov).

## Introduction

Hyperhydration has a detrimental effect on both morbidity and mortality. It increases the risk of acute kidney failure, the need for renal replacement therapy (RRT) and worsens the recovery of renal functions. It also worsens acute lung injury (ALI) and infectious complications, prolongs invasive mechanical ventilation (IMV), and stays in the Intensive care unit (ICU) [1–6].

Real-time assessment of fluid status in critically ill patients is a challenge. Echocardiography can quickly identify hemodynamic phenotypes but is a more intermittent method than a continuous one [7]. Semiinvasive methods, based on monitoring stroke volume using the area under the arterial curve, do not provide information on interstitial fluid. This issue is partially addressed using transpulmonary thermodilution, measuring extravascular lung water (EVLW), and lung ultrasound [6,7,8]. Calculating the cumulative fluid balance (CFB) is inaccurate [10]. The gold standard deuterium dilution methods for total body water (TBW) are not practical [6].

In addition to hyperhydration, rapid muscle loss in critically ill patients due to a severe catabolic state and immobilization worsens the outcome [11,12]. It is difficult to monitor skeletal muscle mass (SMM). Ultrasound is not accurate and requires well-trained staff [13]. Dual energy X-ray absorptiometry (DEXA) provides a reasonably accurate picture of bone mass and soft tissue. However, repeated X-ray examination is not the method of choice [14,15].

Bioelectrical impedance vector analysis (BIVA) is a noninvasive method based on the principle that the flow of alternating electrical current through a particular tissue differs depending on the content of water. It is capable of measuring body composition. Using 50 frequencies of bioimpedance spectroscopy, we can distinguish total body water (TBW), extracellular water (ECW), and calculate intracellular water (ICW) from their difference. However, it cannot distinguish between intravascular and interstitial volume in the extracellular compartment [16–18]. According to several studies, the results of the bioimpedance parameters of body composition are comparable to DEXA [19–21]. An important prognostic parameter is the phase angle (PA), which shows the current time delay of the passage through cell membranes, that is, a phase shift between the sinusoidal voltage waveforms and the current [22–24]. Among laboratory markers, presepsin is significant for prognosis [25,26].

The primary objective of our study was to explore the feasibility of using bioelectrical impedance vector analysis (BIVA) to monitor hydration status and skeletal muscle mass to predict the outcome of critically ill patients (both the ECMO and the non-ECMO group). The secondary objective was to compare the BIVA parameters (overhydration (OHY), ECW/TBW and quadrant, vector length, PA) with the recorded cumulative fluid balance (CFB). Finally, to determine the prognostic ability of the phase angle (PA of BIVA) compared to other prognostic markers (APACHE II, serum presepsin).

## Methods

### Study design

We conducted a prospective, observational, double blinded study (from March 2021 to May 2022) to evaluate hydration status and lean body mass as measured by BIVA in critically ill adult patients admitted to the 24-bed ICU of the University Hospital Ostrava. The study was approved by the Ethics Committee of the Ostrava University Hospital (ref. No. 56/2021). Informed consent was obtained from the person legally responsible for the patient.

### Inclusion criteria

We included patients with respiratory failure of COVID-19 who developed acute respiratory distress syndrome (ARDS), characterized by acute onset, bilateral pulmonary infiltrates, hypoxemia (paO_2_/FiO_2_<300), and with the expectation of at least 7 days of invasive mechanical ventilation (IMV), and/or veno-venous extracorporeal membrane oxygenation.

### Exclusion criteria

We excluded patients with an extremely unfavourable prognosis, a short expected stay in the ICU (APACHE II≥30), metastatic malignancy, post-CPR (cardiac arrest syndrome) conditions before admission, cerebral edema, brain trauma, intracranial hypertension, liver cirrhosis, or pre-existing neurodegenerative disease.

We also excluded patients following limb amputation or patients with pacemakers and implantable defibrillators, conditions that are not compatible with the use of BIVA.

### Study protocol

We enrolled adult patients (≥18 years) within 48 h after admission to the ICU, who were mechanically ventilated and expected to remain in the ICU on the IMV for at least 7 days.

Demographic data was recorded at admission to the ICU (age, sex, APACHE II score, BMI, yes/no COVID, duration of IMV, yes/no ECMO, yes/no RRT, yes/no delirium (CAM-ICU), length of stay in the ICU, length of hospitalization, ICU mortality, length of hospitalization before ICU admission, ICU admission: surgical, trauma, medical).

Regular BIVA measurements were performed in all patients at baseline within 48 h after hospitalization, then following one and two weeks apart (after 7 and 14 days). Depending on the length of hospitalization, 2–3 measurements were made in each patient.

Two well-trained nurses measured fluid status and body composition using bioimpedance spectrometry (BIS Multiscan 5000 CE 0120). This is a device with 50 frequencies of electrical current to distinguish between intra- and extracellular fluid.

The following parameters were evaluated at each measurement:

Hydration parameters (in liters and %): Total body water (TBW), Extracellular water (ECW), Intracellular water (ICW), ECW/TBW ratio, Overhydration (OHY).

Body composition parameters (indices in kg/m^2^, nonindexed values in kg): Active body mass index (ATH), Body mass index (BMI), Fat, Body fat mass index (BFMI), Fat free mass index (FFMI), Skeletal muscle mass (SMM) and Body cell mass (BCM).

Nutritional indicators: basal metabolic rate in kcal (BMR), NUTRIC index (NI), Prediction marker (PM – 200/5 kHz impedance ratio).

BIVA raw parameters: Resistance (R), Reactance (Xc), Phase angle (PA), BIVA vector lengths, BIVA quadrant, point outside the 75^th^ percentile.

Daily monitoring included recording of cumulative fluid balance (CFB) and laboratory tests (albumin, prealbumin, CRP, presepsin). We compared the hydration parameters of BIVA with CFB at the same time points (on the baseline and then at weekly intervals).

Due to a possible large bias in the measured data due possibly to extracorporeal circulation, which can lead to electrical dispersion to the environment, we also performed statistical analyses of the effect of hyperhydration on mortality separately for the ECMO group and the non-ECMO group. There is a lack of evidence in the literature on the feasibility of BIVA in patients with ECMO, so we did not exclude these patients.

### Statistical analysis

We set the statistical significance at a p-value of 0.05. Missing values and outliers were double checked, imputed, labelled, and removed from further statistical processing. Categorical variables were characterized by the absolute count and the relative frequency (in %). Fisher’s exact test was used to assess the statistical difference between the groups. Numeric variables with medians and 25–75 % quartiles and statistically tested with the two-sample nonparametric Wilcoxon rank sum test.

The association of variables with mortality was assessed using the two-sample test mentioned above. Due to the suspicious bias introduced by mixing patients with ECMO and non-ECMO, the analyses were repeated for these groups separately.

## Results

### Description of the study cohort

**Fig. 1:**
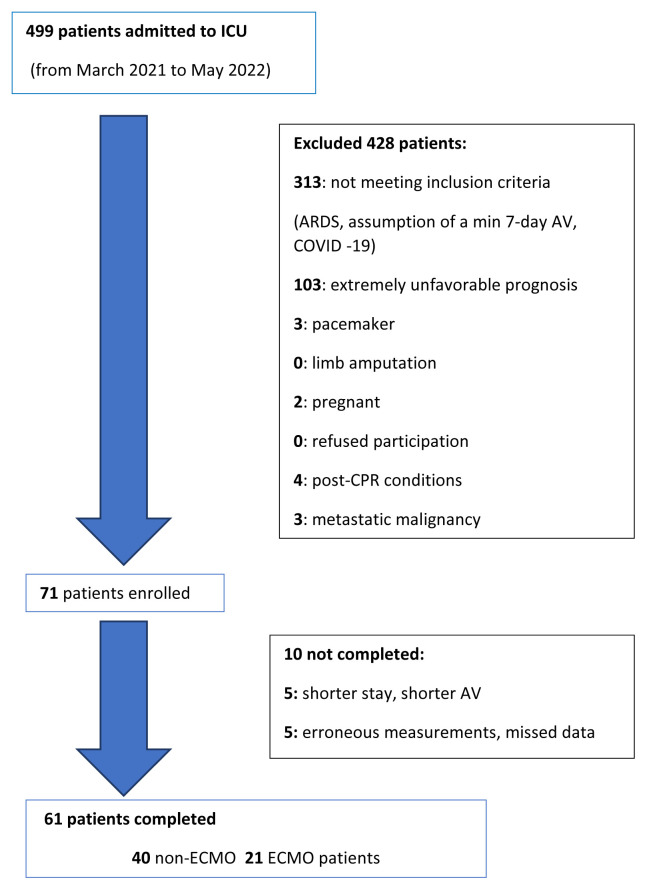
Flow chart. ICU = intensive care unit; ARDS = acute respiratory distress syndrome; AV = artificial ventilation; post-CPR = postcardiac arrest syndrome; ECMO = extracorporeal membrane oxygenation.

A total of 61 patients were enrolled in the study from March 2021 and May 2022. We included patients with respiratory failure who needed invasive mechanical ventilation and/or ECMO. See flow chart in Figure 1.

Given the period in question, these were patients with ARDS. In our group, 37 of 61 patients (61 %) were men and 24 (39 %) were women. The median age was 49 years (45–57 years). During the study, 15 patients died (D) – 8 (53 %) men and 7 (47 %) females. 46 patients survived (S) – 29 (63 %) men and 17 (37 %) women. Neither the gender difference nor the age difference between the D (47 years, 41–50.5) and S (51.5 years, 45.25–57.75) groups was significant.

The main characteristics of the study cohort are shown in Table 1.

### Baseline characteristics

The overall mortality rate in our group of 61 patients with ARDS was 24.59 % (15 of 61 patients). The mortality risk was in line with the severity of the disease. The APACHE II score in the D group (died) was 24 (22–29) compared to 22 (18–24) for the S group (survived), and the difference was statistically significant (p=0.0052). ECMO, with 34 % of patients (21 of 61 patients) dependent on this form of support, also showed a clear association (p=0.0005). Mortality in the ECMO group was 52.38 % (11 out of 21 died), while in the non-ECMO group it was only 10 % (4 out of 40 patients died). The risk of death increased with obesity (median BMI 39.2 in D compared to 30.75 in S). The median BMI in our group of 61 patients with COVID-19 was 31.6 (ranging from a minimum value of 26.38 to a maximum BMI value of 46.85), with a high proportion of fat. Obese patients predominated. In the deceased group, the BMI was higher at 39.2 compared to the survived group at 30.75. However, this is not a statistically significant result given the BMI distribution among individual patients. No decrease in skeletal muscle mass was detected between the data measured at admission and after one and two weeks. However, serum albumin decreased more in the D group (median 31 g/l) compared to the S group (34 g/l); p-value 0.0308. Serum albumin levels depended on the degree of inflammation. CRP levels at admission were 52–160 mg/l, and the difference between the D and S groups was not statistically significant. However, it is worth noting that the CRP at admission was higher in the D group (median 129.1 mg/l) compared to the S group (median 100.8 mg/l), together with a higher drop in albumin after one week of hospitalization – D 31 g/l vs. S 34 g/l.

### BIVA fluid parameters and outcome

BIVA-measured hyperhydration (OHY, ECW/TBW and lower left quadrant, out of the 75 percentile) did not show a clear statistical risk of mortality when both ECMO and non-ECMO patients were taken together (ECW/TW in D 0.5 vs. S 0.48 and OHY in D 4.6 l vs. S 3.3 l). Similarly, neither PA nor BCM showed any prognostic significance. In the D group, PA values were only slightly lower (at intake D 4.6° vs. S 5.0° and after one week D 4.15° vs. S 4.6°). Similarly, the difference in BCM that reflects body cell mass, the metabolically active part of FFM (D 25 vs. S 34.89) was not statistically significant (p=0.0745) and resistance depending on hydration did not show a specific difference. On admission, the statistical significance of the location in the lower left quadrant with the point outside the 75^th^ percentile was very close to the borderline (p=0.0395).

The non-ECMO group consisted of a total of 40 patients with 10 % mortality. We observed a distinct effect of hydration on mortality. In this group, men were predominant with 70 % (28). There were no differences in weight and age between the D and S groups. The statistical significance of the association of the APACHE II score (D 29 vs. S 21) with mortality was clearly maintained here (p=0.047). In this group of non-ECMO patients, hyperhydration showed its influence on mortality, with ECW/TBW increasing more dramatically in the D group [0.56 compared to 0.49 for the S group; p=0.0050] and in OHY [6.9 l in D vs. 3.7 l in S; p=0.0402]. This contrasts with the calculated CFB, where the differences between the groups are unclear. Thus, we can confirm the significantly higher accuracy of the hydration status measured by bioimpedance compared to that inferred by calculating the CFB. According to these findings, there is a more pronounced decrease in BCM – 27.4 for the D group versus 35.9 in the S group; borderline significance (p=0.0583). A prognostic role for the phase angle was also confirmed, with PA decreasing to a median of 3.30° (3.18–3.42) in the D group, while the S group had a higher PA of 4.95° (3.88–6.0); p=0.0051. Of these parameters, ECW/TW and PA have the highest statistical significance for predicting mortality. However, the assumption of a prognostic role (in terms of mortality) for the location of BIVA in the lower left quadrant with the lowest resistance (OHY) and reactance (low BCM) was not proven, as was the case for decreases in SMM; Table 2.

In the ECMO group with 11 dead patients and 10 surviving patients, we observed virtually no effect of the hydration and nutritional parameters measured by BIVA on the prediction of mortality. The only difference is a greater decrease in resistance and reactance after one week in the deceased group, but this was only marginally significant (p=0.0946). In the ECMO group, we did not observed any significant differences in the measured parameters between D and S. Only BMI at admission (D 41.4 vs. S 29.85) with a higher fat content (D 51.3 kg vs. S 26.5 kg) and a greater decrease in albumin after one week (median 31 g/l in the D group vs. 37 g/l in the S group) showed any relationship with mortality.

Among the laboratory markers we monitored (3 measurements in 61 patients), we observed that the levels of presepsin are higher and that of albumin and prealbumin are lower in group D. Furthermore, the increase in presepsin in the D group (from 1250 to 1650 pg/ml in one week) is greater compared to the S group (521 to 836 pg/ml). The decrease in albumin at 30.5 g/l and prealbumin at 0.20 g/l was more significant in the D group compared to the smaller decrease in the S group (albumin 34.0 g/l and prealbumin 0.34 g/l). However, laboratory data, higher baseline CRP (D 129 vs. S 58.4) or higher presepsin (D 1072 vs. S 988 pg/ml) in the D group, did not show statistically significant difference; see Table 3. The statistical significance of these differences counts cannot be determined, probably due to the small sample size and the large variance; see the Table S1.

We also analysed the dependence of laboratory markers on BIVA parameters that characterize hyperhydration (short vector length – location in the lower left quadrant, low PA, high ECW/TBW ratio, ICW) along with possible confounders (gender, age, BMI and ECMO organ support). These variables were measured repeatedly, so we were able to collect sufficient data even from a relatively small number of patients. The results were analyzed using mixed linear regression models that are suitable for repeated measurements. To evaluate their dependence on bioimpedance, we chosed linear models for albumin (a pathophysiologically related to inflammation, capillary leakage, and fluid leakage into the interstitium) and presepsin (with its prognostic role in critically ill patients). For each dependent variable (albumin, presepsin), two different models were used, each containing confounders (ECMO, sex, age and BMI) as follows:

- BIVA + PA
- ICW + ECW/TW

As the models are fitted on normalised data, the β coefficient estimates they produced can be interpreted as effect sizes of the corresponding variables; Table 4.

Presepsin showed a positive dependence on the ECMO support (β=0.41, p=0.0666), on age (increasing with age, β=0.31, p=0.0160) and on the length of the BIVA vector (the lower the vector length, the higher the presepsin, β=0.37, p=0.0021). This reflects the dependence of hyperhydration (shorter BIVA vector, i.e. location in the 1st left lower quadrant) and increased presepsin, confirming hyperhydration as an unfavorable prognostic marker. The relationship between presepsin and PA was not confirmed. The linear model of the relationship between presepsin and hydration (ECW/TW), showed a β of 0.52 (p=0.0004), confirming the association of the increase in presepsin with hyperhydration, as expressed by the increase in ECW/TW and ICW (β=0.65, p=0.0002), with a concurrent effect of ECMO (β=0.42, p=0.0327), age (β=0.24, p=0.0194) and BMI (β=0.37, p=0.0007).

Albumin showed the same dependence on age (decreasing albumin with increasing age, β=-0.39, p=0.0062) and on the length of the BIVA vector (shorter BIVA in hyperhydration, lower albumin; β=0.29, p=0.0213). We also confirmed an inverse relationship between albumin level and hydration, that is, a decrease in albumin is associated with an increase in ECW/TBW (β=-0.41, p=0.0074) and an increase in ICW (β=-0.53, p=0.0050), with a concurrent effect of age (β =-0.34, p=0.0031) and BMI (β=-0.26, p=0.0271).

## Discussion

Intravenous fluid administration is one of the most widely used interventions in emergency care. Properly used, these interventions can save lives, but the wrong amount or type of fluid can have serious consequences [1–6]. Depending on the severity of the condition, pro-inflammatory activity, and glycocalyx damage, fluid extravasation may occur [5,6]. Covid patients are very sensitive to hyperhydration due to a high degree of SIRS [27]. Hyperhydration and muscle wasting are known to be risk factors for mortality [28,29]. To prevent the detrimental effects of hyperhydration on organ function, proper monitoring of hydration status, the potential development of edema, and the accumulation of ECW remain considerable challenges in clinical practice. BIVA is a suitable method for real time assessment of hydration status and body composition [28,30]. Although there are still too many uncertainties in the interpretations of BIVA in critically ill patients, especially in patients with organ support and/or extracorporeal methods [31].

The key results of our study are that we were able to demonstrate the feasibility of BIVA in critically ill patients on organ support. Above all, we prove that the parameters of the BIVA fluid can predict mortality in critically ill patients, but only in patients without ECMO support. However, BIVA did not show an obvious loss of skeletal muscle during the critical catabolic condition.

### 1. Feasibility of BIVA to monitor hydration status

BIVA is a rapid, noninvasive bedside technique to assess TBW, ECW, and body composition. We can measure hyperhydration as the ECW/TW ratio (norm 0.36–0.39) or OHY, which is the difference between the amount of ECW detected by BIVA and the predicted ECW due to weight and height under the euvolemic condition [32].

The measurement of hydration by BIVA has previously been reported to be feasible and useful in various clinical conditions such as heart and kidney failure, dialysis, and malnutrition [33,34]. There are still discrepancies regarding the interpretation of this technique in critically ill patients. BIVA precision may be affected by rapid fluid transfers during circulatory fluid resuscitation, by consequences of organ support (RRT, ECMO), and thus its interpretation becomes less straightforward. BIVA has been validated compared to dilution methods, which are considered the gold standard [35,36], although there have been suggestions that BIVA can be reliably used to assess hydration status, including among the critically ill [35–37].

We established a prospective observational clinical study in 61 critically ill COVID-19 patients where we took 2–3 consecutive BIVA measurements (depending on the length of stay). We found that BIVA is feasible and safe, in addition to being rapid, noninvasive, and repeatable. Measurements could be made even in patients with organ support during their stay in the ICU. The reproducibility of the measurements was very good, but body weight must be known, so weighing procedures are still necessary.

The reproducibility of BIVA was very good under all conditions during ICU monitoring and artificial ventilation in the Dewitte study with 25 critically ill patients and 3 consecutive daily BIVA measurements [38]. The three consecutive BIVA measurements in our study were each one week apart. In both studies a new 50 frequencies BIS technology was used. This study provides evidence for an improved correlation between daily changes in TBW (BIVA) and fluid balance after excluding patients with extracorporeal circulation. Similarly, our results demonstrate an improved correlation between hyperhydration (increased ECW/TW and decrease in PA) and mortality after excluding patients with ECMO.

On a close examination of the BIVA studies in critically ill patients [37–39], we see that they include only a small number of patients with ECMO support. Patients on ECMO support were absent in the Yang study [39]. Of the 25 critically ill patients in the Dewitte study [38], only 10 were in the extracorporeal circulation (RRT or/and ECMO). There are studies with patients who receive CRRT during real-time fluid status using BIVA but not ECMO support; Chen [20] with 89 mixed ICU patients and Rhee [37] with 208 patients with AKI. Both studies confirm the suitability of BIVA during CRRT.

Of the 61 critically ill patients in our study, the ECMO cohort comprised 21 patients and 11 patients were on CRRT. However, in the ECMO cohort, we did not observe significant differences in measured parameters between the D and S groups, raising the question whether bioelectrical impedance is a suitable method to obtain valid data on hydration status from patients with extracorporeal circulation.

### 2. The Effect of BIVA-measured hyperhydration on mortality

Several studies in medical/surgical patients have reported high statistical significance for the association between elevated hydration status (ECW/TBW, OHY, reactance, resistance), decreased PA measured by BIVA, and mortality [39–47]. Combining BIVA (ECW/TW, OHY) with brain natriuretic peptide (BNP) levels can be a strong predictor of acute decompensated heart failure [40,41]. The ECW/ICW ratio allows discrimination of survivors from non-survivors [43–45].

Similarly, a decrease in PA was significantly correlated with fluid overload and a worse prognosis. Hyperhydration increases mortality in all categories of patients, prolongs the stay in the ICU, increases the need for mechanical ventilation and/or RRT, can increase the incidence of infectious complications and worsens the recovery from organ dysfunction [1–6].

In critically ill patients in our study who required invasive mechanical ventilation, but without the need for ECMO, we observed a correlation between overhydration and mortality. Specifically, we observed a higher increase in ECW/TW in the deceased (0.56 in D vs. 0.49 in S) and a higher rate of OHY (median of 6.9 l in D vs. 3.7 l in S). Similar conclusions were reached in a study of 140 critically ill patients, when an increase in hydration volume on day 3 in the ICU was associated with a higher risk of in-hospital mortality [39]. Another aspect in which this study agrees with our observations is the confirmation of a higher prognostic role for hydration measured by BIVA compared to the calculated cumulative balance. Also in our study, bioimpedance-measured hyperhydration demonstrated its effect on mortality with statistical significance (p=0.0050), the calculated cumulative balance showed a wide variance in both the D and S groups (-2095 to 6395 ml) and did not show statistically significant differences.

The lack of correlation between mortality and survival in the ECMO group and the phase angle can be explained by the fact that ventilation issues outweigh hemodynamics difficulties.

### 3. Comparison of the prognostic ability of the PA (BIVA) with that of other prognostic markers (APACHE II, serum presepsin) and loss of muscle mass

Bioimpedance-derived muscle mass (skeletal muscle mass, fat free mass) appears to be a promising biomarker for sarcopenia, correlated with computed tomography (CT) and DEXA [30,48,49]. Body cell mass (BCM) and FFM have the potential to estimate metabolic rate, protein requirements, and pharmacokinetics [36]. The phase angle expresses the relationship between the reactance and resistance, both raw parameters. The greater the number of cell membranes through which the electrical signal must pass through, the greater the reactance, and thus the PA. In this way, PA serves as an important prognostic factor, with a normal value between 4–15°, because altered fluid balance and malnutrition are characterized by changes in cell membrane integrity [23,24]. Low PA is associated with increased morbidity, nutritional risk, and frailty [23].

We were able to demonstrate the prognostic ability of PA in our group of non-ECMO patients. Among the patients who died (in the D group), PA decreased to a median of 3.30° (3.18–3.42), while in the S group (patients who survived) PA remained higher at 4.95° (3.88–6.0). Therefore, together with the ECW/TW ratio that reflects overhydration, it showed the strongest statistical significance in predicting mortality, p-value 0.0051. However, we could not confirm the effect of a decrease in SMM or location in BIVA in the left lower quadrant with the lowest resistance (overhydration) and reactance (low BCM) on mortality.

Loss of muscle mass and muscle function leads to ICU-acquired weakness (ICU-AW), a serious complication that significantly worsens the prognosis of critically ill patients, both in the short term (inability to wean from invasive mechanical ventilation, significant slowing of rehabilitation) and in the long term. It most often results in Post-Intensive Care Syndrome (PICS), persistent difficulties after severe illness in the muscular, mental, and cognitive domains. However, we did not observe a statistically significant decrease in SMM in our group of patients with hyperinflammation in IMV, leading to the assumption of rapid development of ICU-AW. The explanation may lie in timing, since the first two BIVA measurements occur within the first week of hospitalization. ICU-AW develops initially from a loss of muscle strength and then only leads to a loss of muscle mass. This is mainly due to perturbations in excitation-contraction coupling (altered calcium homeostasis, disrupted contractile actin/myosin interactions, decreased membrane excitability) and mitochondrial dysfunction. But first of all, changes in body water correspond to changes in muscle mass. Water represents approximately 76 % of muscle mass with a reduction in the elderly. ICW depletion may produce an intracellular catabolic signal, proteolysis. Loss of ICW with age partially explained the loss of muscle mass and muscle strength, known as sarcopenia [50]. On the other hand, an increase in ECW, interstitial swelling, can overestimate SMM. An increase in the ECW/ICW ratio>1 is associated with a poorer prognosis [50]. As Moonen points out, intramuscular edema is assessed by both BIA and CT as a muscle mass [31].

When selecting laboratory markers related to hydration, nutritional status and mortality, we chose those that play a prognostic role in critically ill patients, such as albumin, prealbumin (inflammation-dependent serum proteins) and presepsin (soluble portion of the cluster differentiation 14 subtype (sCD14-ST). It is a glyco-protein expressed in the membranes of monocytes and macrophages in response to damage-associated molecular patterns (DAMPs) or infection, pathogen-associated molecular patterns (PAMPs) [25]. In our measurements of 61 patients, presepsin levels were higher, while albumin and prealbumin levels were lower, in the D group. However, there was a large variance in the data and the difference was not statistically significant. Linear models of the dependence of albumin and presepsin on bioimpedance hydration data were chosen to demonstrate the association. Regression analysis confirms the correlation between hyperhydration (short BIVA vector length and higher ECW/TW ratio) and increased presepsin with a concomitant decrease in albumin. These data confirm the adverse effect of hyperhydration on mortality, seen as increased presepsin correlated with an increased ECW/TW ratio and shortening of the BIVA vector, that is, location in the left lower quadrant.

### 4. BIVA-guided the management of fluid therapy

Despite BIVA’s ability to monitor trends in hydration, assess the risk of hyperhydration, and serve as a prognostic marker, its application to guide the management of fluid therapy in critically ill patients remains limited. Serial BIVA measurements can help establish an optimal target weight in patients undergoing chronic dialysis, thereby preventing complications during fluid removal [51]. A randomized controlled trial involving 65 critically ill patients undergoing continuous renal replacement therapy (CRRT) investigated the effectiveness of BIVA-guided fluid removal [52]. BIVA-guided ultrafiltration (UF) was associated with a higher UF rate and increased urine output; however, it did not increase the incidence of hypotension or the need for vasopressors. Although fluid overload was less frequent, there were no differences in duration of ventilatory support or mortality compared to the control group. Similar findings were reported in a study of patients with sepsis-associated acute kidney injury (AKI) who received CRRT [53]. Currently, BIVA serves only as a supportive tool in the management of fluid therapy, particularly in unstable patients with sepsis who experience rapid fluid changes. In addition, larger and better-designed studies are needed to evaluate the impact of BIVA-guided fluid therapy on patient survival, as well as kidney and other organ recovery [31].

## Limitations

A limitation of this study is the small sample size in one center: of 61 patients, 15 died and 46 survived. To mitigate the risk of bias, we divided the data set into ECMO and non-ECMO patients, resulting in 11 deceased and 10 surviving patients in the ECMO group and 4 deceased and 36 surviving patients in the non-ECMO group. Unfortunately, due to the data sample, only the largest differences between D and S were shown to be statistically significant. However, the Wilcoxon rank sum test remains appropriate for such small samples. Unfortunately, some methods, such as the evaluation of receiver operating characteristic (ROC) curves, are not suitable in this context. On the other hand, we have used also mixed models, which rely on repeated measurements, provide more data samples, and are therefore suitable.

## Conclusions

Clinical trials such as this are necessary to determine the feasibility and validity of using BIVA in critically ill patients with organ support, including extracorporeal membrane oxygenation. We were able to demonstrate hyperhydration measured by BIVA (ECW/TBW and OHY) and low phase angle (PA) to predict the outcome in the group of critically ill non-ECMO patients. The ECMO support limits the accuracy of the BIVA method.

## Supplementary Information

**Supplementary Table 1:** Laboratory markers.

	Died	Survived	All	p-value
*N*	15	46	61	
*Albumin [g/l]*	31.00 (29.50–34.50)	35.00 (31.00–37.00)	34.00 (30.00–37.00)	0.0031
*Prealbumin [g/l]*	0.22 (0.16–0.35)	0.23 (0.13–0.34)	0.23 (0.13–0.35)	NS
*Presepsin [pg/ml]*	1000.50 (693.75–1729.75)	585.00 (365.00–1095.00)	664.00 (402.00–1221.00)	0.0014
*CRP [mg/l]*	96.00 (17.15–137.70)	54.50 (22.80–110.00)	59.55 (20.85–127.53)	NS

Median (25–75 % quantile), p-value: Wilcoxon rank sum test. NS = not significant.

## Figures and Tables

**Table 1:** Baseline characteristics.

	Died	Survived	All	p-value
*N*	15	46	61	
*Sex:*				NS
• *female*	7 (46.67 %)	17 (36.96 %)	24 (39.34 %)	
• *male*	8 (53.33 %)	29 (63.04 %)	37 (60.66 %)	
*Age*	47.00 (41.00–50.50)	51.50 (45.25–57.75)	49.00 (45.00–57.00)	NS
*Weight [kg]*	108.91 (90.89–134.59)	97.02 (83.19–110.15)	99.96 (84.31–114.98)	NS
*APACHE II*	24.00 (22.00–29.00)	22.00 (18.00–24.00)	22.00 (19.00–25.00)	0.0052
*ECMO*	11 (73.33 %)	10 (21.74 %)	21 (34.43 %)	0.0005
*RRT*	5 (33.33 %)	6 (13.04 %)	11 (18.03 %)	NS
*Active body mass index [%]*	73.40 (61.00–77.55)	70.55 (61.15–84.27)	70.60 (61.00–83.30)	NS
*BMI [kg/m* *^2^* *]*	39.20 (29.60–46.85)	30.75 (26.38–37.93)	31.60 (28.10–39.50)	0.0491
*Fat [kg]*	38.60 (25.15–53.20)	26.75 (17.08–39.22)	28.20 (17.30–45.90)	NS
*Body fat mass index [kg/m* *^2^* *]*	10.80 (8.60–21.20)	7.85 (5.32–13.35)	9.40 (5.70–15.50)	NS
*Fat free mass index [kg/m* *^2^* *]*	24.60 (22.75–26.00)	22.25 (20.12–24.77)	23.30 (20.50–25.70)	0.0845
*Skeletal muscle mass SMM [kg]*	36.70 (31.10–41.30)	35.55 (30.02–44.87)	35.80 (30.40–43.60)	NS
*Body cell mass [kg]*	33.40 (28.70–37.20)	34.85 (28.82–42.58)	33.90 (28.70–41.40)	NS
*Basal metabolic rate [kcal]*	1948.00 (1794.00–2243.50)	2006.00 (1760.50–2295.00)	1996.00 (1760.00–2260.00)	NS
*Nutritional index*	0.50 (0.46–0.55)	0.48 (0.44–0.51)	0.49 (0.45–0.52)	NS

Categorical variables: count (percentage), p-value: Fisher’s exact test; numerical variables: median (25–75 % quantile), p-value: Wilcoxon rank sum test. APACHE = Acute physiology and chronic health evaluation; ECMO = extracorporeal membrane oxygenation; RRT = renal replacement therapy; BMI = body mass index; SMM = Skeletal muscle mass; NS = not significant.

**Table 2:** The non-ECMO group.

	Died	Survived	p-value
*N*	4	36	
*Sex:*			NS
• *female*	1 (25.00 %)	11 (30.56 %)	
• *male*	3 (75.00 %)	25 (69.44 %)	
*Age*	56.00 (48.00 – 64.00)	54.00 (46.00 – 59.50)	NS
*Weight [kg]*	95.83 (91.29 – 102.23)	99.01 (83.68 – 114.23)	NS
*APACHE II*	29.00 (26.75 – 29.25)	21.00 (18.00 – 24.00)	0.0417
*ECMO*	0 (0.00 %)	0 (0.00 %)	NS
*RRT*	2 (50.00 %)	4 (11.11 %)	0.0997
*TBW*	44.30 (41.08 – 48.20)	48.35 (43.68 – 55.82)	NS
*ECW*	24.00 (23.15 – 25.82)	24.35 (20.90 – 26.13)	NS
*ICW*	20.30 (17.92 – 22.38)	24.45 (21.90 – 29.68)	0.0678
*OHY*	6.90 (6.07 – 8.88)	3.70 (1.55 – 6.10)	0.0402
*CFB [ml]*	315.00 (−262.50 – 1615.00)	320.00 (−1195.00 – 940.00)	NS
*ECW/TBW*	0.56 (0.55 – 0.57)	0.49 (0.44 – 0.51)	0.0050
*Active body mass index [%]*	73.55 (69.60 – 77.08)	71.65 (62.23 – 84.62)	NS
*BMI [kg/m* *^2^* *]*	32.55 (29.65 – 37.45)	31.30 (27.12 – 38.65)	NS
*Fat [kg]*	25.15 (20.70 – 32.65)	26.75 (16.48 – 39.82)	NS
*Body fat mass index [kg/m* *^2^* *]*	8.60 (6.83 – 12.20)	8.20 (4.92 – 13.25)	NS
*Fat free mass index [kg/m* *^2^* *]*	24.25 (23.47 – 24.88)	22.85 (20.42 – 24.97)	NS
*Skeletal muscle mass [kg]*	36.25 (33.03 – 40.72)	36.45 (31.08 – 45.32)	NS
*Cell mass [kg]*	27.40 (26.05 – 28.85)	35.90 (30.18 – 42.33)	0.0583
*Basal metabolic rate [kcal]*	2013.50 (1870.25 – 2144.50)	2017.50 (1798.00 – 2327.25)	NS
*Nutritional index*	0.56 (0.55 – 0.57)	0.48 (0.44 – 0.51)	0.0050
*Prediction marker*	0.88 (0.87 – 0.89)	0.82 (0.78 – 0.86)	0.0130
*ALBUMIN 1*	30.50 (29.25 – 32.00)	34.00 (29.75 – 38.25)	NS
*PREALBUMIN*	0.11 (0.07 – 0.15)	0.11 (0.08 – 0.16)	NS
*PRESEPSIN*	1250.50 (846.00 – 1641.25)	836.00 (523.25 – 1258.50)	NS
*C-reactive protein*	95.65 (53.72 – 130.98)	108.35 (60.20 – 164.02)	NS
*Phase angle*	3.30 (3.18 – 3.42)	4.95 (3.88 – 6.00)	0.0051
*RESISTANCE*	347.33 (334.74 – 362.36)	378.64 (326.28 – 453.22)	NS
*REACTANCE*	19.11 (18.62 – 20.69)	32.84 (26.00 – 45.55)	0.0109
*BIVA*	214.23 (210.22 – 218.23)	216.00 (182.00 – 259.37)	NS
*BIVA quad:*			NS
• *dehydration*	0 (0.00 %)	0 (0.00 %)	
• *anasarca*	2 (100.00 %)	25 (86.21 %)	
• *obese*	0 (0.00 %)	4 (13.79 %)	
*BIVA outside 75 % 1 quad*	1.00 (1.00 – 1.00)	1.00 (0.00 – 1.00)	NS

Categorical variables: count (percentage), p-value: Fisher’s exact test; numerical variables: median (25–75 % quantile), p-value: Wilcoxon rank sum test. APACHE = Acute physiology and chronic health evaluation; ECMO = extracorporeal membrane oxygenation; RRT = renal replacement therapy; TBW = total body water; ECW = extracellular water; ICW = intracellular water; OHY = overhydration; CFB = cumulative fluid balance; BMI = body mass index; BIVA = bioelectrical impedance vector analysis; NS = not significant.

**Table 3:** ECMO group.

	Died	Survived	p-value
*N*	11	10	
*Sex:*			NS
• *female*	6 (54.55 %)	6 (60.00 %)	
• *male*	5 (45.45 %)	4 (40.00 %)	
*Age*	47.00 (39.00 – 47.00)	47.50 (41.00 – 50.75)	NS
*Weight [kg]*	114.98 (96.48 – 136.59)	91.02 (80.56 – 100.63)	0.0448
*APACHE II*	24.00 (22.00 – 26.00)	23.00 (22.00 – 24.75)	NS
*ECMO*	11 (100.00 %)	10 (100.00 %)	NS
*RRT*	3 (27.27 %)	2 (20.00 %)	NS
*TBW*	43.00 (38.30 – 54.25)	48.70 (45.07 – 52.93)	NS
*ECW*	21.10 (18.70 – 23.65)	21.85 (20.52 – 23.60)	NS
*ICW*	22.40 (18.50 – 27.95)	26.30 (23.73 – 27.45)	NS
*OHY*	3.10 (1.05 – 5.45)	2.50 (1.18 – 5.00)	NS
*CFB [ml]*	50.00 (−395.00 – 3455.00)	630.00 (−177.50 – 1805.00)	NS
*ECW/TBW*	0.49 (0.45 – 0.50)	0.47 (0.44 – 0.48)	NS
*Active body mass index [%]*	63.50 (61.00 – 77.55)	65.25 (61.15 – 74.95)	NS
*BMI [kg/m* *^2^* *]*	41.40 (32.35 – 49.80)	29.85 (25.90 – 34.10)	0.0528
*Fat [kg]*	51.30 (28.10 – 56.50)	26.50 (20.68 – 37.20)	0.0980
*Body fat mass index [kg/m* *^2^* *]*	15.50 (9.70 – 22.90)	7.50 (6.53 – 13.05)	0.0980
*Fat free mass index [kg/m* *^2^* *]*	25.20 (22.55 – 26.45)	20.80 (19.42 – 23.65)	NS
*Skeletal muscle mass [kg]*	36.70 (31.10 – 41.30)	30.70 (26.62 – 38.35)	NS
*Cell mass [kg]*	33.80 (32.10 – 38.95)	34.30 (26.82 – 43.55)	NS
*Basal metabolic rate [kcal]*	1948.00 (1794.00 – 2243.50)	1851.00 (1686.75 – 2151.75)	NS
*Nutritional index*	0.49 (0.45 – 0.50)	0.47 (0.44 – 0.49)	NS
*Prediction marker*	0.82 (0.80 – 0.85)	0.82 (0.80 – 0.84)	NS
*ALBUMIN*	33.00 (30.50 – 35.00)	34.50 (29.25 – 37.00)	NS
*PREALBUMIN*	0.13 (0.06 – 0.16)	0.13 (0.10 – 0.18)	NS
*PRESEPSIN*	1072.00 (866.50 – 1744.00)	988.00 (544.50 – 1258.00)	NS
*C-reactive protein*	129.10 (117.90 – 168.25)	58.40 (38.70 – 120.07)	NS
*Phase angle*	5.30 (4.35 – 5.85)	5.05 (4.68 – 5.77)	NS
*RESISTANCE*	340.41 (299.54 – 381.45)	418.72 (334.76 – 509.36)	NS
*REACTANCE*	31.77 (24.12 – 42.03)	36.41 (29.90 – 47.50)	NS
*BIVA*	205.77 (179.16 – 227.40)	238.26 (205.12 – 280.42)	NS
*BIVA quad:*			NS
• *dehydration*	0 (0.00 %)	1 (12.50 %)	
• *anasarca*	9 (81.82 %)	6 (75.00 %)	
• *obese*	2 (18.18 %)	1 (12.50 %)	
*BIVA outside 75 % 1 quad*	1.00 (1.00 – 1.00)	1.00 (0.75 – 1.00)	NS

Categorical variables: count (percentage), p-value: Fisher’s exact test; numerical variables: median (25–75 % quantile), p-value: Wilcoxon rank sum test. APACHE = Acute physiology and chronic health evaluation; ECMO = extracorporeal membrane oxygenation; RRT = renal replacement therapy; TBW = total body water; ECW = extracellular water; ICW = intracellular water; OHY = overhydration; CFB = cumulative fluid balance; BMI = body mass index; BIVA = bioelectrical impedance vector analysis; NS = not significant.

**Table 4:** Linear mixed regression models.

*Model*	Variable	Estimate	Std. Error	p-value
*Albumin (BIVA + PA + confounders)*	(intercept)	0.03	0.17	NS
	ECMO	0.00	0.24	NS
	sex = woman	−0.11	0.29	NS
	age	−0.39	0.13	0.0062
	BMI	0.02	0.14	NS
	BIVA	0.29	0.12	0.0213
	PA	−0.05	0.10	NS

*Albumin (ICW + ECW/TBW + confounders)*	(intercept)	0.06	0.14	NS
	ECMO	−0.10	0.21	NS
	sex = woman	−0.04	0.25	NS
	age	−0.34	0.11	0.0031
	BMI	−0.26	0.11	0.0271
	ICW	−0.53	0.18	0.0050
	ECW/TBW	−0.41	0.15	0.0074

*Presepsin (BIVA + PA + confounders)*	(intercept)	−0.13	0.16	NS
	ECMO	0.41	0.21	0.0634
	sex = woman	0.14	0.26	NS
	age	0.30	0.12	0.0185
	BMI	0.04	0.13	NS
	BIVA	−0.39	0.12	0.0012
	PA	0.03	0.10	NS

*Presepsin (ICW + ECW/TBW + confounders)*	(intercept)	−0.06	0.13	NS
	ECMO	0.42	0.19	0.0327
	sex = woman	0.03	0.23	NS
	age	0.24	0.10	0.0194
	BMI	0.37	0.10	0.0007
	ICW	0.65	0.17	0.0002
	ECW/TBW	0.52	0.14	0.0004

ECMO = extracorporeal membrane oxygenation; BMI = body mass index; BIVA = bioelectrical impedance vector analysis; PA = phase angle; ICW = intracellular water; ECW/TBW = extracellular water/total body water.
